# Evaluation of Retention of two Different Cast Post-Core Systems and Fracture Resistance of the Restored Teeth

**Published:** 2015-06

**Authors:** Amir Ali Reza Khaledi, Shekufe Sheykhian, Arash Khodaei

**Affiliations:** 1 Dept. of Prosthodontic, School of Dentistry, Shiraz University of Medical Sciences, Shiraz, Iran;; 2 Students’ Research Committee, School of Dentistry, International Branch, Shiraz University of Medical Sciences, Shiraz, Iran;

**Keywords:** Pulp less teeth, Fracture resistance, Retention, Cast metallic post-core

## Abstract

**Statement of the Problem:**

The survival of pulpless teeth restored with different post and core systems is still a controversial issue.

**Purpose:**

This study compared the retention of two different post and core systems and also the fracture resistance of teeth restored with these systems.

**Material and Method:**

Eighty endodontically treated maxillary central incisors were sectioned perpendicular to the long axis at a point 2mm incisal to the cemento-enamel junction (CEJ) and then the root canals were obturated.

The restored teeth were randomly divided into two equal groups of 40. One group was restored with Nickel-Chromium (Ni-Cr) post and core system and the other group with Non-Precious Gold alloy (NPG) system. For evaluation of fracture resistance of the restored teeth, the specimens (n=20 per each group) were mounted in acrylic resin blocks and a layer of polyvinyl siloxane was applied to cover the roots. Loads were applied at an angle of 45 degrees to the long axis of the teeth and measured with a universal testing machine.

The axial retention values of the studied groups (no=20) were measured on an Instron testing machine at a crosshead speed of 0.5 mm/min. Statistical analyses were performed using SPSS version 19.00 and student’s t-test (α=0.05).

**Results:**

Although retention failure load for Ni-Cr system was lower than NPG system, there was no significant difference between the two systems (*p*= 0.7). However, fracture resistance of the teeth restored with Ni-Cr post and core system was significantly higher than NPG group (*p*= 0.000).

**Conclusion:**

There was no significant difference between the retention of the studied post and core systems. Although significantly higher fracture thresholds were recorded for Ni-Cr post and core group, the failure loads of both systems may rarely occur clinically.

## Introduction

Dental practitioners are often faced with the task of restoring endodontically treated teeth. Root canal treatment is usually the consequence of caries followed by pulpal infection or traumatic damage to a tooth. Trauma and caries are mostly associated with an extensive loss of tooth structure.[1]

The pulpless tooth has already lost substantial coronal tooth structure from the access preparation for the endodontic treatment[2] which necessitates restoration of the tooth with a complete crown for esthetic and functional rehabilitation. When a large portion of the clinical crown has been lost to damage, it is often impossible to achieve optimum retention of a restoration in the remaining dentin.[1-2] Therefore the type of restoration of endodontically treated teeth is closely related to the amount of the tooth structure that remains after endodontic therapy.[2] The factors to be considered when restoring endodontically treated teeth are the role of moisture loss, the nature of dentin, alteration in strength caused by architectural changes in morphology of teeth, concepts of biomechanical behavior of tooth structure under stress, and changes in the collagen alignment.[3-7]

Reinforcement of endodontically treated or structurally weakened teeth prior to placement of extra coronal restorations becomes necessary if desirable prognosis for the restoration is expected. Recent studies have confirmed clinical observations of increased fracture rate in endodontically treated teeth.[5-7] These conditions often call for the construction of an intraradicular foundation to prevent the fracture and to help support the crown restoration.[6-8] Various methods for rebuilding endodontically treated teeth have been introduced to the professionals. They vary from custom-made dowel-core systems to several simplified one-visit techniques in which prefabricated dowel and/or pins and composite resin or amalgam are used.[8]

Many investigators have reported that the design and the material of the post and core affect the resistance to fracture of endodontically treated teeth restored with post and core systems.[7-11] The cast gold alloy dowel and core has been regarded as the gold standard for foundation restorations.[10-11] Because of its very good properties such as high biocompatibility, high corrosion resistance, and high rigidity, it has been used extensively in the past, but currently its use has diminished due to the high cost of gold.[12-13] Cast post and core, when compared with prefabricated post and core, are suggested to be used in non-radicular root canals because of their accurate adaptation to the remainder tooth structure.[14-16] Cast post is capable of resisting rotational forces, has superior success rate, and can be easily removed to permit endodontic retreatment.[10-11,17]

Currently, the material of choice for custom-made cast-metal dowel-core system is Ni-Cr alloy. Due to high rigidity of Ni-Cr posts, less reduction of tooth structure is needed so that the maximum retention and fracture resistance of the post will be provided.[7, 17]

In the study conducted by Hayashi *et al.*,[18] the teeth restored with Ni-Cr cast posts had significantly higher fracture resistance than other groups and had the lowest risk of vertical root fracture. Also, Ni-Cr alloys create a layer of chromium oxide that resists tarnish. These alloys have also some disadvantages, for example, most of them have a breaking point over half of the root length due to the high post stiffness. If these teeth become fractured, they are unrepairable.[13-16] The other disadvantages are difficulty in finishing and polishing processes, doubt in biocompatibility due to presence of nickel and subsequent allergic reactions,[13] and absence of physical characteristics similar to dentin. However, in spite of these disadvantages, it still has the highest usage in fabricating post-cores.[18]

Controversial results have been reported from *in vitro* researches in regard to various post and core system. For instance, Dakshinamurthy *et al.*[19] indicated that the highest fracture resistance was recorded with Ni-Cr cast post and core in comparison with prefabricated titanium post and core. Maccari *et al.*[20] showed that the teeth restored with cast posts had fracture strength twice as high as the teeth restored with resin post. Assif *et al.*[21] made a comparison between the fracture resistance of the teeth restored with cast post-cores and those restored with various types of post designs and did not detect any significant differences. McDonald *et al.*[22] compared the fracture resistance of the teeth restored with a cast post, teeth restored with a carbon-fiber post, and intact root-treated teeth (controls); they found no significant differences among them.[23] De Castro Albuquerque *et al.*,[24] Amussen *et al.*[25] and Lanza *et al.*[26] reported that glass fiber and cast post and cores had similar fracture resistance regardless of the type of the tooth. Trabert *et al.*[27] concluded that conservation of tooth structure enhanced resistance to fracture regardless of the design of the post. Hoag and Dwyer[28] determined that a complete cast crown on extracted molars was more important than the type of post and core technique for preventing fracture. However, Kern *et al.*[29] noted no statistically significant improvement in sheer strength of post-reinforced extracted molars when the crowns were implemented.[30]

A new alloy containing more than 80% copper called Non-Precious Gold alloy (NPG) has been introduced. It is claimed that although NPG alloy has optimum mechanical and physical properties to act as post, the preparation and trimming of this alloy is much easier than Ni-Cr.[31-32] Due to the lack of research about NPG alloy and any comparison with other post and core systems, this study aimed to make a comparison between the retention and fracture resistance of the teeth restored with the most common and popular post and core system fabricated from Ni-Cr and those restored with the new introduced NPG post and core.

## Materials and Method

In this experimental study, 80 maxillary central incisors, extracted within the two months before study, were selected from a total of 500 teeth. The teeth had shown an intact crown without caries, restoration, previous root canal therapy (RCT), crack and any attrition with comparable length and diameter. Very long or very short teeth with severe curve were excluded from study. The mean length of the roots was 15.40±0.53 and the mesiodistal width was 6.17±0.41. Fiber-optic transillumination was used to inspect the roots for fracture lines. Radiography in two buccolingual and mesiodistal dimensions was used for detection of any calcification, internal resorption, open apex and accessory canal; and the teeth that had these issues were excluded. Each tooth was given a number (from 1 to 80). Using SPSS 19.0 software, the teeth were randomly allocated into two groups (n=40). Group A was restored with dowels and cores fabricated using Ni-Cr and group B was restored with dowel and cores fabricated by using NPG alloy. The composition, characteristics and manufacturer of each alloy is presented in Table 1.

**Table 1:** The alloys used in this study

**Alloys**	**Composition**	**Modulus of** **Elasticity (MPa)**	**Vickers** **Hardness HV1**	**Elongation** **Percent %**	** Compression/ Density g/cm[3] **	**Manufacturer**
Ni-Cr	Ni (61.4%), Cr (25.7%), Mo (11%), Si (1.5%), Mn (less than 1%), Al (less than 1%), Cl (less than 1%)	200GPa	235HV1	12%	8.24 gr/cm[3]	Ivoclar Vivadent Germany
NPG	Cu (80.7%), Al (7.8%), Fe (3%), Zn (2.7%), Mn (1.7%), Ni (4.3%)	not available	140HV1	15%	7.8 gr/cm[3]	Aalbadent USA

Ni-Cr= Nickel-Chromium, NPG= non-precious gold alloy

The teeth were immersed in 5% sodium hypochlorite for 15 minutes in order to remove the organic materials from the root surfaces. Any remaining tissue was carefully cleaned by using a periodontal curette and then stored in distilled water. 

The crowns were cut into horizontal sections, perpendicular to the long axis at a line 2mm incisal to the most coronal point of the proximal cementoenamel junction (CEJ), using diamond discs mounted on a lathe-cut machine under continuous water coolant.

*Mounting the Teeth in Acrylic Blocks*

The specimens were individually mounted vertically in self-cure acrylic resin in the root block former (2×2×2cm^3^) at the level of CEJ. In the 40 samples used for evaluation of fracture resistance, PDL had to be simulated so the teeth were dipped into a molten wax to a depth of 2mm below CEJ to provide a 0.2 to 0.3 mm spacer before being embeded in the resin. Acrylic resin was poured into the root block before and after observing the first signs of polymerization, then the teeth were removed from the resin blocks. The wax was eliminated and replaced by polyvinyl Siloxane impression material (Zhermack; Elite HD, Italy) injected into the acrylic resin alveolus. They were then reinserted into the acrylic block. During the course of polymerization, the acrylic resin block was cooled in water to avoid dehydration of the dentin and also to prevent the deformation of resin. The roots were prepared to have 2mm ferruled collar with 5mm diameter, 6 convergences, and a 1mm shoulder finish line.

*Root canal preparation and obturation*

After preparing the access cavity by use of a high speed air motor (NSK; Tochigi, Japan), the working length was established 1 mm shorter than the apex. The canals were instrumented to working length with size 40 K-flex file (Mani; Tochigi, Japan). A step back flaring technique was performed at 1mm increments with Gates Glidden burs (Mani; Tochigi, Japan) number 2-3. A size 15 K-flex file was passed through the apical foramen of the canal before and after instrumentation to ensure patency. The root canal was irrigated with 15ml of 1.25% NaOCl after every file change. The root canals were dried with sterile paper points (Orca; Tiagin, China) before filling. A size 40 gutta-percha (Gapadent; Haburg, Germany) master cone coated with ZOE sealer (Dorident Vienna, Ausrtia) , was inserted into the canal. Root canals were obturated using lateral condensation technique with finger spreader (Mani; Tochigi, Japan). Finally, excess gutta-percha was removed and condensed with a hot plugger.

*Dowel- hole preparation*

A dowel hole of 11mm length was standardized and prepared by peeso2, 3 (Mani; Tochigi, Japan). Before drilling, excess gutta-percha was removed by Gates Glidden drills. The canals were cleaned by using air/water spray and then dried by paper points.

*Impression technique*

Fabrication of post and cores can be categorized as direct or indirect technique. However impression technique was used for duplicating the dowel hole. It had two stages as following: primary impression was taken by a partial plastic tray filled with putty. After removing a thin layer of putty at the sites of the tooth, additional light silicone was injected into the canal by a special syringe and excess material was transported by lentulo (Mani, Tochigi, Japan) to the canal. Then hairpins as impression post was inserted into the canal. Finally, impression was taken by putty tray.

*Post and core fabrication method*

The casts were poured with type IV die stone (Whip mix; Dortmond, Germany). Post and core models were waxed up and burned out identically into two groups. Group A was injected with Ni-Cr and group B with NPG. In specimens for evaluating retention, a1mm diameter hole width was prepared in the core at the stage of wax up which was duplicated in the final core. The length of the core in all samples was accurately measured and made uniform at 4mm.

*Dowel cementation*

The dentin walls of the dowel space were etched with 37% phosphoric acid (Denfil etchant- Gangowon , Korea) for 40 seconds to eliminate the effect of sealer, and then rinsed and gently air dried. The dowel was coated with glass ionomer (GI) cement (GC; Tokyo, Japan) and mixed according to manufacturer’s instructions. GI is based on the reaction of silicate glass powder and polyalkenoic acid. Typical percentages of the powder materials are: silica (41.9%), alumina (28.6%), aluminum fluoride (1.6%), calcium fluoride (15.7%) and aluminum phosphate (3.8%). Also, cement was transported into the canal by lentulo. After the posts were inserted gently to reduce hydrostatic pressure, they were positioned in place under firm finger pressure and the excess cement was removed. Then the samples were kept in normal saline for a week in a refrigerator.

*Placing specimens on the measuring machine (Instron Testing Machine)* ******

Before placing the samples on the device, using SPSS 19.0 software, each of the groups A and B was divided into two 20-teeth groups. The fracture resistance was measured in one group and retention in the other one. In order to measure fracture resistance and retention, Universal Instron Testing Machine (Zwick-Roell; GmbH, Germany) was used. The device was calibrated before placing the samples. The position and the direction of the samples in the machine were set by the device itself. The specimens were placed in a customized, self-aligning apparatus, which was clamped into place with a vise grip. When assembled, the horizontal rod attached to the upper element of the Instron testing machine was passed through the hole which was made in the core. The acrylic holder allowed the teeth to be hold firmly during retention testing. To measure the amount of retention, shear force was applied to the cement with speed of 0.5 mm/min. Force was applied until the post was removed from the canal. The force required to remove the post from the canal along its longitudinal axis was reported in Newton to show the amount of retention. To measure the fracture resistance, the samples were inserted into the device at a 45-degree angle to the long axis of the teeth and compressive load was applied to them with a speed of 0.5 mm/min. Maximum force that caused each of samples to break down was reported in Newton as the amount of fracture resistance. Mean fracture strength and retention were analyzed using SPPS version 19.00 and Students t-test at the significant level of 0.05. 

## Results

**Figure 1:**
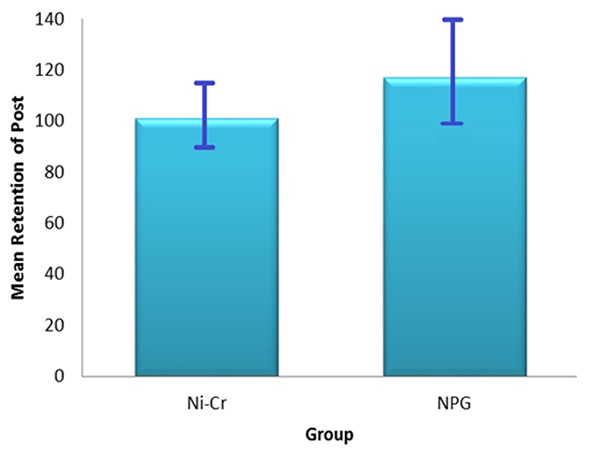
Retention values of the studied groups

**Figure 2:**
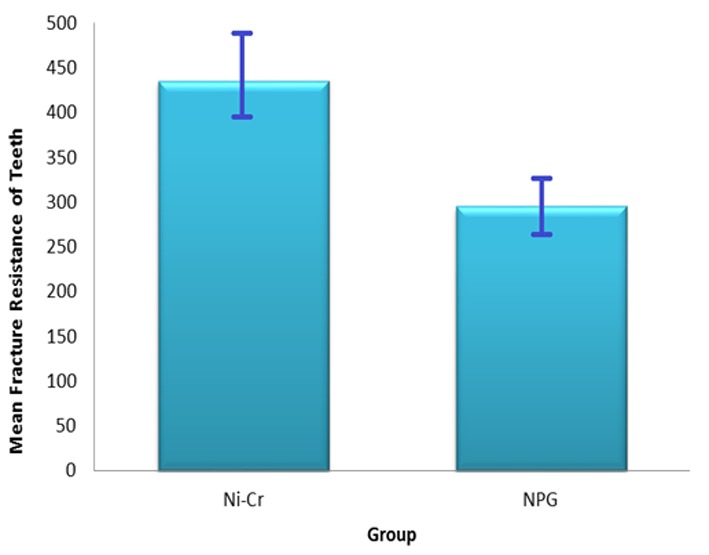
Fracture resistance values of the teeth restored with the studied post-core systems

Fracture resistance of the restored teeth and retention of posts were measured using an Instron testing machine. Data were evaluated statistically using Independent t-test. The mean and standard deviation values for fracture resistance of restored teeth have been summarized in Table 2 and Figure 1; values for post retention are shown in Table 3 and Figure 2. Although the mean retention of Ni-Cr system (101.01 N) was lower than NPG (117.02N), statistical analysis revealed no significant difference in regard to retention of the studied dowel-core systems (*p*= 0.70).

**Table 2:** Mean, standard deviation and P-value of retention of the post and core systems

**Variable**	**Dowel** **systems**	**Number of** **Specimen**	**Mean**	**SD**	***p*** ** value**
Retention of post	Ni-Cr	20	101.01	16.26	0.70
	NPG	20	117.02	20.50	

**Table 3:** Mean, standard deviation and P-value of fracture resistance of the restored teeth

**Variable**	**Dowel** **systems**	**Number of** **Specimen**	**Mean**	**SD**	***p*** **value**
Fracture resistance of teeth	Ni-Cr	20	435.60	52.19	0.000
	NPG	20	295.80	26.56	

Comparing the fracture resistance of restored teeth revealed that the posts in Ni-Cr group demonstrated higher mean fracture strength and this difference was statistically significant (*p*< 0.001).

None of the specimens was broken down from core portion or either core-post interface. 

## Discussion

The results of the present study showed that there was a statistically significant difference between the fractures resistances of the teeth restored with Ni-Cr and those restored with NGP alloys; however, the retention of the two groups was relatively the same.

In this *invitro* study, the teeth extracted were carefully selected regarding standard size and quality. Nevertheless, considerable variations were observed in the fracture resistance of the extracted teeth. Similar variations occurred in all experimental groups, however by reviewing the literature, it was revealed that the use of extracted teeth for this study was valid. Attempts were made to simulate the periodontal ligaments and tooth-supporting structures; thus, the roots were not embedded directly into the resin blocks. The thin layers of polyvinyl siloxane simulated periodontal ligaments, the acrylic resin simulated alveoli and blocks were used to simulate bony sockets. By not embedding the roots directly into the acrylic resin blocks, external reinforcement of the root structure by the rigid acrylic resin was avoided. Since external rigid reinforcement of the root is not normally found in oral cavity, therefore; it may alter the strength of the roots and consequently the patterns of failure. The mean size of the roots was 15.40±0.53mm in length, and 6.7±0.41mm in mesiodistal width. Attempts were made to calibrate the canal preparations. The matching-size twist drill was used to prepare the post space for each specimen. To minimize variations in lengths of the posts, the teeth were carefully selected to ensure similar root lengths. In the current study, all specimens were restored and tested without complete-coverage crowns. The placement of a crown during endodontic restoration testing has been questioned, as this practice may obscure the effects of different buildup techniques.[33-34] The crown creates a ferrule effect and different load distribution when placed over a core buildup if the margins encircle a sound dentin collar.[7, 35-37] In this study, the test loads were applied directly on the cores, not to artificial crowns. If complete crowns with 2-mm ferrules were included, the results of this study might have been different.[37-38] Root canal instrumentations were simulated, and canal obturation procedures were accomplished, although canal obturation should have little or no effect on the strength of the roots.[2, 27, 39-40]

It has been suggested that a post should have the same modulus of elasticity as root dentin to distribute the applied forces evenly along the length of the post.[17, 41] However, Creugers *et al.*[11] reviewed the related literature (studies that have been published in more than 20 years) and reported that survival rates have varied largely in endodontically treated teeth restored with different post-and-core systems. No consensus existed on which technique and materials are best suited for use;[9, 11, 42] some studies reported significantly higher mean failure loads for fiber posts,[9] or significantly higher mean failure loads for metal posts.[43-44]

In the current study, the greatest numbers of fractures were reported in the group restored with NPG alloy. This result may be attributable to the high modulus of elasticity of Ni-Cr posts. Higher modulus of elasticity results in less bending of the post/core unit under load; consequently, less stress and more force distribution are exerted on the tooth. This phenomenon was also reported in previous investigations.[7, 45] It has been suggested that the stiffness of the dowel in an endodontically treated tooth compromised by lost tooth structure can be a reinforcing medium. The results of Sidoli *et al.*[46] showed that the fracture strength of the teeth with carbon fiber dowels was lower than that for teeth with metal dowels. Since the dowels used in the present study had the same cross-sectional areas and shapes, the bending stiffness of the dowels would be directly proportional to the modulus of elasticity of each material.[12]

Despite these measures, several factors limit the direct application of this study to *in-vivo* situations; for example, mechanical and thermocycling procedures were not used. Simulated clinical conditions might have affected the results; further studies that simulate the oral environment are recommended. Moreover, although all the teeth with cast posts and cores failed as a result of tooth fractures, the results could have been different if complete crowns with 2-mm ferrules had been cemented over the cast cores.

In the current study, the retentive capacity of the two groups of dowel and core systems was compared by subjecting them to tensile loading. The result of our study concerning the retention showed that retentive values for NPG dowels were higher than those of Ni-Cr dowels and cores but the difference was not statistically significant. Laboratory studies have investigated the retention of various post systems and the variables reported to have affected retention included length, diameter and design of the post, canal shape and preparation, luting medium, method of cementation,[47] and location in the dental arch.[48] Since in this study all variables affecting post retention were the same, the retentive property of the two tested groups did not differ from each other significantly.

The data obtained from this study corresponds well to those found by other investigators in that the surface configuration of a dowel is the most important variable in retention.[14, 49] It was shown that the retentive strength of the reinforced composite resin dowel-core systems may be increased significantly by roughening the dentinal walls or creating undercuts into the walls of the channel. Addition of pins may also promote the retention and resistance of this system.[8, 49-50]

Retention and resistance to fracture are two important factors that must be achieved with post-and-core retained restorations. Nevertheless, retention often requires the removal of tooth structure, a procedure that may reduce the strength of the root. When placing a post, the dentist must evaluate each tooth individually to determine the best approach for obtaining the maximal fracture resistance. Because a single post system is unlikely to satisfy retentive requirements for all clinical situations, a variety of post systems are suggested to achieve the optimal balance between post retention and resistance to root fracture. This flexible approach should allow the dentist to successfully restore most endodontically treated teeth. 

## Conclusion

Teeth restored with Ni-Cr post and core system showed higher resistance to fracture compared with the group restored with NPG post and core system.NPG post and core system showed higher retention to tooth structure than Ni-Cr cast and core. Both post and core systems showed acceptable clinical behavior. 
